# Gut Microbiota Metabolite Indole Propionic Acid Targets Tryptophan Biosynthesis in *Mycobacterium tuberculosis*

**DOI:** 10.1128/mBio.02781-18

**Published:** 2019-03-26

**Authors:** Dereje Abate Negatu, Yoshiyuki Yamada, Yu Xi, Mei Lin Go, Matthew Zimmerman, Uday Ganapathy, Véronique Dartois, Martin Gengenbacher, Thomas Dick

**Affiliations:** a Department of Medicine, Yong Loo Lin School of Medicine, National University of Singapore, Singapore, Republic of Singapore; b St. Peter TB Specialized Hospital, Addis Ababa, Ethiopia; c Department of Pharmacy, Faculty of Science, National University of Singapore, Singapore, Republic of Singapore; d Public Health Research Institute, New Jersey Medical School, Rutgers, The State University of New Jersey, Newark, New Jersey, USA; e Department of Microbiology & Immunology, Yong Loo Lin School of Medicine, National University of Singapore, Singapore, Republic of Singapore; Colorado State University; Max Planck Institute for Infection Biology

**Keywords:** NTM, TrpE, allosteric inhibitor, antibiotic, tryptophan mimic

## Abstract

Indole propionic acid (IPA), produced by the gut microbiota, is active against Mycobacterium tuberculosis *in vitro* and *in vivo*. However, its mechanism of action is unknown. IPA is the deamination product of tryptophan (Trp) and thus a close structural analog of this essential aromatic amino acid. *De novo* Trp biosynthesis in M. tuberculosis is regulated through feedback inhibition: Trp acts as an allosteric inhibitor of anthranilate synthase TrpE, which catalyzes the first committed step in the Trp biosynthesis pathway. Hence, we hypothesized that IPA may mimic Trp as an allosteric inhibitor of TrpE and exert its antimicrobial effect by blocking synthesis of Trp at the TrpE catalytic step. To test our hypothesis, we carried out metabolic, chemical rescue, genetic, and biochemical analyses. Treatment of mycobacteria with IPA inhibited growth and reduced the intracellular level of Trp, an effect abrogated upon supplementation of Trp in the medium. Missense mutations at the allosteric Trp binding site of TrpE eliminated Trp inhibition and caused IPA resistance. In conclusion, we have shown that IPA blocks Trp biosynthesis in *M. tuberculosis* via inhibition of TrpE by mimicking the physiological allosteric inhibitor of this enzyme.

## Abstract (precis)

New drugs against tuberculosis are urgently needed. The tryptophan (Trp) analog indole propionic acid (IPA) is the first antitubercular metabolite produced by human gut bacteria. Here, we show that this antibiotic blocks Trp synthesis, an *in vivo* essential biosynthetic pathway in *M. tuberculosis*. Intriguingly, IPA acts by decoupling a bacterial feedback regulatory mechanism: it mimics Trp as allosteric inhibitor of anthranilate synthase, thereby switching off Trp synthesis regardless of intracellular Trp levels. The identification of IPA’s target paves the way for the discovery of more potent TrpE ligands employing rational, target-based lead optimization.

## INTRODUCTION

The human microbiota produces a multitude of molecules, including nonribosomal peptides, thiopeptides, lantibiotics, bacteriocin, and amino acid metabolites that play numerous roles in microbe-microbe and microbe-host interactions (1–5). Recently, the host microbiota has been shown to influence early lung colonization by Mycobacterium tuberculosis (6). We have discovered that the gut microbiota metabolite indole propionic acid (IPA) (7, 8) inhibits growth of M. tuberculosis *in vitro* and in a mouse model of infection (9). Whether a link exists between IPA-producing bacteria in the gut and tuberculosis lung disease remains to be determined (6, 9–11).

The antibacterial mechanism of action of this endogenously produced natural product has not been determined. IPA is the deaminated form of the aromatic amino acid l-tryptophan (Trp) (8). *M. tuberculosis* is capable of synthesizing Trp *de novo*. Zhang et al. (12) and Wellington et al. (13) have demonstrated that the Trp biosynthetic pathway is essential for growth and viability of *M. tuberculosis* in standard (Trp-free) medium as well as in macrophages and mice. In *M. tuberculosis*, Trp regulates its own synthesis by acting as a cooperative allosteric inhibitor of anthranilate synthase TrpE, which catalyzes the first committing step in the pathway (14, 15).

The homodimeric *M. tuberculosis* protein TrpE (Rv1609), in conjunction with glutamine amidotransferase (TrpG) providing ammonia, catalyzes the formation of anthranilate from chorismate (15, 16). The N-terminal region of TrpE contains the allosteric Trp binding motif _53_LLESX_10_S_67_ (15). The 10-amino-acid residue X_10_ loops of the two TrpE subunits approach each other at the interface, providing part of the structural framework for the cooperative allosteric inhibitory effect of Trp binding. In addition, TrpE residues _170_HHEGT_174_ are involved in the cooperative allosteric inhibition mechanism by making interactions with their subunit’s Trp binding motif as well as with the Trp binding motif and the _170_HHEGT_174_ residues of the partner subunit (15).

In view of the structural relationship between IPA and Trp, we hypothesized that IPA may exert its antibacterial activity by mimicking Trp. Here we present metabolic, chemical rescue, genetic, and biochemical analyses showing that IPA exerts its whole-cell anti-*M. tuberculosis* activity by blocking Trp biosynthesis through allosteric inhibition of TrpE activity.

## RESULTS

### IPA docks into the allosteric Trp binding pocket of TrpE.

**FIG 1:**
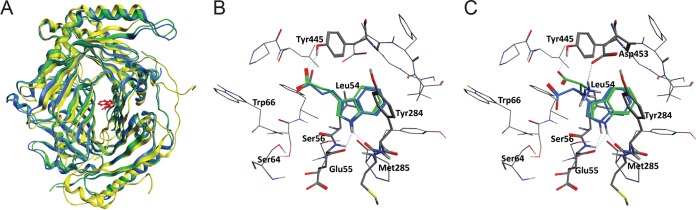
IPA and Trp docking into the allosteric pocket of *M. tuberculosis* TrpE. (A) Superposition of three TrpE crystal structures from *M. tuberculosis* without bound Trp (PDB no. 5CWA [yellow]) (15), Serratia marcescens with bound Trp (PDB no. 1I7S [green]) (19), and *Salmonella* Typhimurium with bound Trp (PDB no. 1I1Q [blue]) (14). The location of Trp is indicated in red. The superposition was performed using the MOE software (44). IPA (B) and Trp (C) were docked into the structure of *M. tuberculosis* TrpE (5CWA) (15) using the AutoDockTools 4 (ADT) and Vina programs (17, 18). The ADT and the Vina poses are colored green and blue, respectively. The calculated binding energies were −5.79 (ADT) and −8.2 (Vina) kcal/mol for IPA and −6.73 (ADT) and −8.2 (Vina) kcal/mol for Trp. Amino acid residues shown in panels B and C have at least one atom within a 5-Å radius of IPA or Trp. Leu54, Glu55, Ser56, Ser 64, and Trp66 are part of the allosteric Trp binding motif _53_LLESX_10_S_67_ (Fig. 5) reported by Bashiri et al. (15).

**FIG 2:**
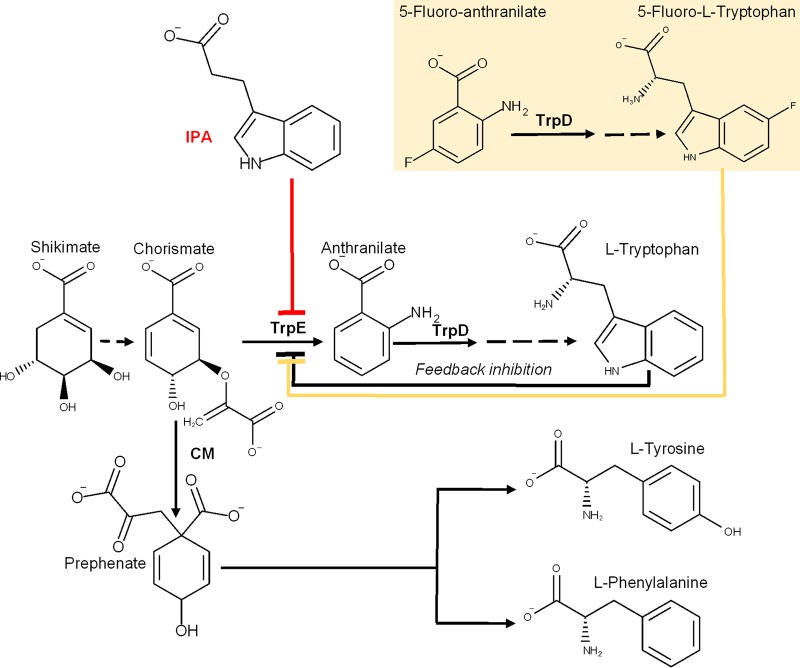
Trp biosynthetic pathway and proposed mechanism of action of IPA. Negative-feedback inhibition by Trp and the proposed inhibitory activity of IPA on anthranilate synthase TrpE are indicated. CM, chorismate mutase. (Inset) The known Trp biosynthesis inhibitor fluoro-anthranilate is a substrate of TrpD and is converted to fluoro-tryptophan (12, 20).

To determine whether IPA can theoretically bind to the allosteric Trp binding pocket of *M. tuberculosis* TrpE, docking experiments were carried out using Autodock4 and Vina software (17, 18). The allosteric binding site for *M. tuberculosis* Trp was determined by superposition using the Trp-bound TrpE structures of Serratia marcescens and Salmonella enterica serovar Typhimurium with the Trp-free structure of *M. tuberculosis* TrpE (14, 15, 19) (Fig. 1A). Figure 1B shows that IPA can be docked into the Trp binding site of *M. tuberculosis* TrpE with a conformation similar to Trp, as shown in Fig. 1C. The indole moieties of IPA and Trp overlap similarly with the Trp binding site, forming hydrogen bonds with the carbonyl groups of Glu55 and/or Met285 and hydrophobic interactions with Leu54 and Tyr284. The positively charged amino group of Trp forms a hydrogen bond with the carboxylate group of Asp453, whereas the carboxylate of Trp is not involved in hydrogen bonding (Fig. 1C). In contrast, the carboxylate of IPA forms a hydrogen bond with the hydroxyl group of Tyr445 (Fig. 1B). The finding that IPA docks into the Trp binding pocket lends support to our hypothesis that IPA mimics Trp as an allosteric inhibitor of TrpE (Fig. 2).

### IPA treatment reduces intrabacterial Trp levels.

**FIG 3:**
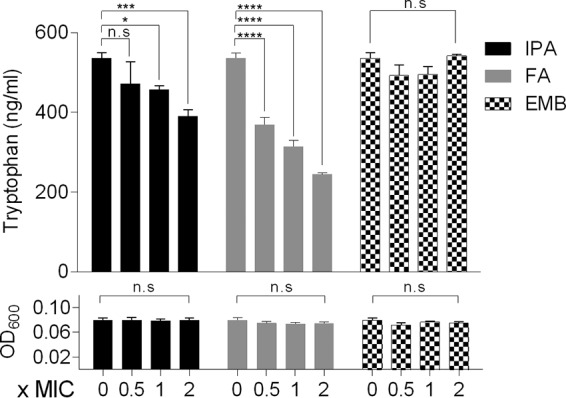
Effect of IPA on intrabacterial Trp concentrations. Exponentially growing M. bovis BCG cultures were treated with increasing IPA, fluoro-anthranilate (FA [positive control]), or ethambutol (EMB [negative control]) concentrations for 24 h, and Trp content was measured using LC-MS. Drug concentrations are given as fold MIC. Experiments were carried out three times independently. A statistical test was performed to determine the significance of Trp reduction upon 24 h of treatment employing a two-way analysis of variance (ANOVA) multiple-analysis tool comparing each treated sample with untreated samples using GraphPad Prism 6 software. n.s, *P* > 0.05; *, *P* ≤ 0.05; ***, *P* ≤ 0.001; ****, *P* ≤ 0.0001.

If the mechanism of action of IPA involves interference with Trp biosynthesis, IPA treatment of *M. tuberculosis* cultures should cause a reduction of the intrabacterial concentration of Trp (Fig. 2). To measure the effect of IPA treatment on intrabacterial Trp levels, we used the biosafety level 2 (BSL2)-compatible surrogate for *M. tuberculosis*, Mycobacterium bovis BCG. The sequences of the TrpE orthologs in both bacteria are identical, and as demonstrated in *M. tuberculosis*, growth of M. bovis BCG is inhibited by IPA (Table 1). Fluoro-anthranilate (FA) was used as a positive control (12). This analog of the Trp pathway substrate anthranilate is converted to fluoro-tryptophan (F-Trp) by TrpD and subsequent pathway enzymes (20), and F-Trp has been shown to allosterically inhibit TrpE (21–23) (Fig. 2). Exponentially growing M. bovis BCG cultures were treated with increasing IPA concentrations, and Trp levels were measured using liquid chromatography-mass spectrometry (LC-MS). Figure 3 shows that IPA reduced Trp levels in a dose-dependent manner. Dose-dependent reduction of Trp was also observed with the positive control fluoro-anthranilate. Reduction of Trp levels by IPA and fluoro-anthranilate at growth-inhibitory concentrations appears to be specific (as opposed to an epiphenomenon of growth inhibition) as these effects were not observed with the antitubercular agent ethambutol (EMB) targeting cell wall synthesis (Fig. 3). This result suggests that IPA interferes with Trp biosynthesis.

**TABLE 1:** Selection and characterization of fluoro-anthranilate- and IPA-resistant M. bovis BCG and *M. tuberculosis* mutants

Selection drug for bacteria^*a*^	Mutation frequency^*b*^	Resistance (no. of strains)^*c*^	Strain	Resistance to:				Mutation(s) in^*d*^:						
				FA		IPA		*trpE*		Rv0880		Rv0948c		Other genes
				MIC (μM) for WT→ mutant	Fold shift	MIC (μM) for WT→ mutant	Fold shift	Nuc	AA	Nuc	AA	Nuc	AA	
FA														
BCG	2 × 10^−8^	FA^r^, IPA^r^ (2)	BCG_FA^R^_M1	7→798	114.0	62→522	8.4	199 (T→G)	67 (S→A)					
			BCG_FA^R^_M2	7→801	114.4	62→518	8.4	388 (G→T)	130 (V→F)					
* M. tuberculosis*	8 × 10^−9^	FA^r^, IPA^r^ (1)	MTB_FA^R^_M1	26→1,350	51.9	112→1,167	10.4	508 (C→A)	170 (H→N)					

IPA														
BCG	7 × 10^−6^	FA^s^, IPA^r(L)^ (6)	BCG_IPA^R^_M1	7→7	0.9	62→167	2.7			196 (A→C)	66 (T→P)			ND
			BCG_IPA^R^_M2	7→8	1.1	62→175	2.8			107 (A→C)	36 (Q→P)			ND
			BCG_IPA^R^_M3	7→6	0.9	62→162	2.6			179 (T→C)	60 (V→A)			ND
			BCG_IPA^R^_M4	7→8	1.1	62→160	2.6			191 (C→T)	64 (S→L)			ND
			BCG_IPA^R^_M5	7→7	1.0	62→182	2.9			231 (T→Δ)	Shift			ND
			BCG_IPA^R^_M6	7→8	1.1	62→398	6.4			191 (C→T)	64 (S→L)			ND
		FA^r(L)^, IPA^r^ (1)	BCG_IPA^R^_M7	7→570	81.4	62→650	10.5					230 (T→C)	77 (V→A)	ND
* M. tuberculosis*	2 × 10^−6^	FA^s^, IPA^r(L)^ (4)	MTB_IPA^R^_M1	26→35	1.3	112→625	5.6			−7 (C→G)	Upstream			
			MTB_IPA^R^_M2	26→31	1.2	112→501	4.5			110 (T→C)	37 (L→P)			
			MTB_IPA^R^_M3	26→24	0.9	112→557	5.0			110 (T→C)	37 (L→P)			
			MTB_IPA^R^_M4	26→27	1.0	112→463	4.1			248 (A→G)	83 (H→R)			
		FA^r(L)^, IPA^r^ (2)	MTB_IPA^R^_M5	26→240	9.2	112→867	7.7					190 (T→C)	64 (S→P)	Rv2019
			MTB_IPA^R^_M6	26→250	9.6	112→1,119	10.0					300 (G→C)	100 (R→S)	Rv3169
* M. tuberculosis* 200 expt	9 × 10^−6^	FA^r^, IPA^r^ (4)	MTB_IPA^R^_M7	26→1,000	38.5	112→809	7.2	271 (C→T)	91 (P→S)					
			MTB_IPA^R^_M8	26→1,400	53.8	112→1,089	9.7	509 (A→G)	170 (H→R)					
			MTB_IPA^R^_M9	26→1,380	53.1	112→1,114	9.9	509 (A→G)	170 (H→R)					
			MTB_IPA^R^_M10	26→1,421	54.7	112→1,009	9.0	509 (A→G)	170 (H→R)					
		FA^r(L)^, IPA^r^ (1)	MTB_IPA^R^_M11	26→300	11.5	112→1,297	11.6					98 (G→T)	33 (R→L)	Rv2942
		FA^s^, IPA^r(L)^ (7)	MTB_IPA^R^_M12	26→33	1.3	112→752	6.7			86 (C→A)	Stop			Rv1576c
			MTB_IPA^R^_M13	26→22	0.8	112→778	6.9			86 (C→G)	Stop			Rv3626c
			MTB_IPA^R^_M14	26→30	1.2	112→704	6.3			98 (C→T)	33 (S→L)			
			MTB_IPA^R^_M15	26→25	1.0	112→671	6.0			101 (T→Δ)	Shift			
			MTB_IPA^R^_M16	26→28	1.1	112→690	6.2			106 (C→T)	Stop			
			MTB_IPA^R^_M17	26→27	1.0	112→605	5.4			281 (T→G)	94 (V→G)			Rv1248c, Rv3626c
			MTB_IPA^R^_M18	26→29	1.1	112→724	6.5			328 (C→T)	Stop			Rv0907

a FA, fluoro-anthranilate; IPA, indole propionic acid. In the experiment listed as “*M. tuberculosis* 200 expt,” 200 IPA-resistant *M. tuberculosis* strains were isolated and then tested for cross-resistance against FA by streak-out on FA-containing agar. Five IPA-FA cross-resistant strains were identified and characterized further. Furthermore, 7 IPA-resistant–FA-sensitive strains from this screen were selected and characterized further.

b Mutation frequency, spontaneous resistance mutation frequency.

c FA^r^, high-level FA resistance; FA^r(L)^, low-level FA resistance; FA^s^, FA sensitive; IPA^r^, high-level IPA resistance; IPA^r(L)^, low-level IPA resistance.

d Nuc, location and nature of single nucleotide polymorphism in coding sequence of respective gene; AA, location and nature of amino acid substitution or other effects on coding sequence (stop/nonsense codon, frameshift) associated with respective DNA polymorphism. MTB_IPA^R^_M1 harbored a C-to-G substitution 7 bp upstream of the start codon of Rv0880. Mutations in other genes represent genes other than *trpE*, Rv0880, and Rv0948c for which whole-genome sequencing revealed DNA polymorphisms. All strains shown were subjected to whole-genome sequencing, with the exception of the BCG strains labeled “ND.” All strains were subjected to targeted Sanger sequencing for *trpE*, Rv0880, and Rv0948.

### Supplementation of medium with Trp abrogates the inhibitory activity of IPA.

**FIG 4:**
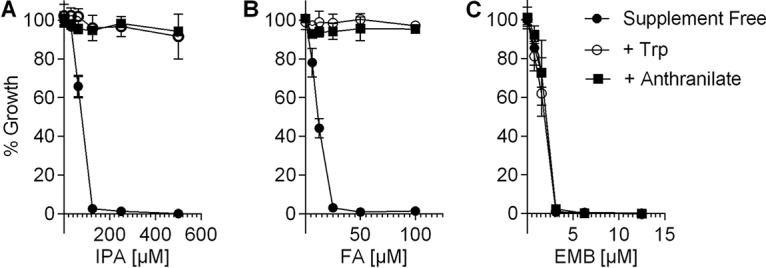
Effect of supplementation of medium with anthranilate or Trp on IPA’s growth-inhibitory activity. Effect of anthranilate and Trp on growth-inhibitory activity of (A) IPA, (B) positive control fluoro-anthranilate (FA), and (C) negative control ethambutol (EMB). Exponentially growing *M. tuberculosis* cultures were treated with increasing concentration of drugs, either without any supplement or with 1 mM Trp or 0.2 mM anthranilate. The OD_600_ was determined after 7 days of incubation in 96-well plates. Experiments were carried out three times independently in duplicate. Mean values and standard deviations are shown.

As the standard mycobacterium broth Middlebrook 7H9 does not contain Trp, *M. tuberculosis* needs to synthesize this amino acid to grow. If IPA acts via blocking Trp synthesis by inhibiting TrpE, exogenous supply of anthranilate or Trp should eliminate the inhibitory effect of IPA (12, 13, 24, 25). *M. tuberculosis* cultures grown in medium without any supplement or with exogenously supplied anthranilate or Trp were treated with increasing IPA concentrations, and the optical density at 600 nm (OD_600_) was measured. Figure 4A shows that the presence of anthranilate or Trp completely suppressed the inhibitory activity of IPA. Figure 4B depicts as a positive control the expected suppressive effect of anthranilate and Trp on the inhibitory effect of fluoro-anthranilate (12). Figure 4C shows that exogenous Trp or anthranilate did not affect the growth-inhibitory activity of the non-Trp pathway targeting ethambutol, suggesting that the effect of the Trp pathway metabolites was specific to inhibitors of the Trp pathway. The results of the medium supplementation experiments were confirmed with a different readout by plating aliquots of culture samples from each treatment condition on 7H11 agar and observing growth on solid medium (see Fig. S1 in the supplemental material). The finding that supplementation of medium with anthranilate or Trp abrogates the inhibitory activity of IPA supports the hypothesis that IPA inhibits synthesis of Trp.

10.1128/mBio.02781-18.2 FIG S1 Effect of supplementation of medium with anthranilate or Trp on IPA’s growth-inhibitory activity measured by spotting experimental drug-treated culture samples on drug-free agar and observing growth. Shown are the effects of anthranilate and Trp on growth-inhibitory activity of IPA, fluoro-anthranilate (FA), and ethambutol (EMB). Exponentially growing *M. tuberculosis* cultures were treated with increasing concentrations of drugs either without any supplement or with 1 mM Trp or 0.2 mM anthranilate. Culture samples were spotted on agar after 7 days of incubation in 96-well plates and grown for 10 days. Experiments were carried out three times independently in duplicate. A representative experiment is shown. Download FIG S1, TIF file, 0.5 MB. Copyright © 2019 Negatu et al. 2019 Negatu et al. This content is distributed under the terms of the Creative Commons Attribution 4.0 International license.

### Mutations at the allosteric Trp binding site of TrpE cause loss of Trp feedback inhibition and resistance to IPA.

**FIG 5:**
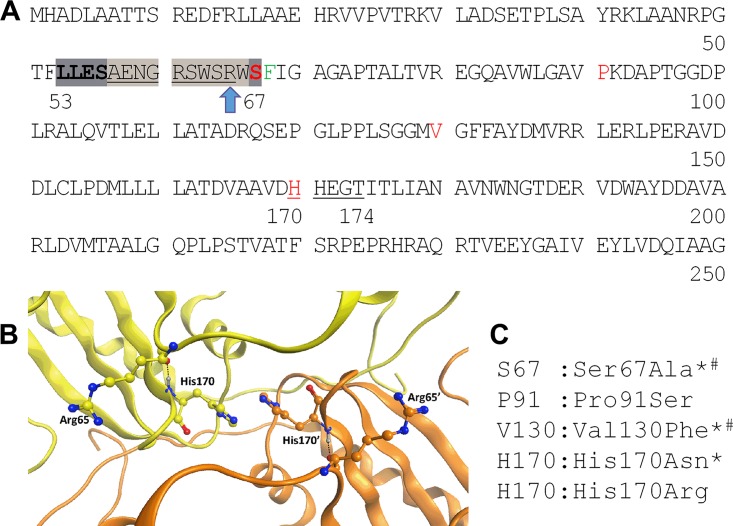
*M. tuberculosis* TrpE amino acid sequence, Trp binding site motifs, and location of IPA–fluoro-anthranilate double-resistant missense mutations. (A) Amino acid sequence of the N-terminal half of TrpE. The allosteric Trp binding motif LLESX_10_S is indicated by the gray background. Residues involved directly in subunit association are underlined. Arg65 in the X_10_ loop of the Trp allosteric binding motif, forming a hydrogen bond with His170, is marked by a blue arrow. Positions of the IPA and fluoro-anthranilate double-resistance mutations identified in this work are indicated in red. The Phe68Ile mutation site identified by Zhang et al. is indicated in green (12). (B) View of the TrpE dimer interface. The two monomers are colored yellow and orange, respectively. Hydrogen bonds are shown as black dashed lines. The amino groups of His170/His170′ form hydrogen bonds with the carbonyl groups of Arg65/Arg65′ (the N···O distance is 2.81 Å). His170 and His170′ interact via pi-pi stacking. Structure and motif annotations are according to Bashiri and colleagues (15). (C) Location and nature of IPA–fluoro-anthranilate double-resistance-conferring missense mutations. *, mutants were selected on fluoro-anthranilate-containing agar. Other mutants were selected on IPA-containing agar. #, mutations identified in M. bovis BCG. Other mutations were derived from *M. tuberculosis*. The TrpE amino acid sequences of M. bovis BCG and *M. tuberculosis* are identical.

If IPA mimics Trp as an allosteric TrpE inhibitor, mutations in the Trp binding site that result in loss of feedback inhibition by Trp should cause resistance to IPA. Zhang and colleagues have shown that resistance to fluoro-anthranilate (which is converted to F-Trp by the mycobacterial Trp synthesis pathway (20) (Fig. 2) emerges via mutations at the Trp allosteric binding site of TrpE. Furthermore, the authors provided direct biochemical evidence that the fluoro-anthranilate resistance mutation Phe68Ile located immediately downstream of TrpE’s allosteric Trp binding motif, _53_LLESX_10_S_67_ (Fig. 5A), eliminated inhibition of TrpE by Trp (12).

To generate *trpE* mutants that have lost Trp binding, we employed a chemical genetic approach and selected mutants with spontaneous resistance to fluoro-anthranilate. M. bovis BCG and *M. tuberculosis* cultures were plated on agar supplemented with fluoro-anthranilate, and three resistant colonies, two from M. bovis BCG and one from *M. tuberculosis*, emerging at a frequency of about 10^−8^/CFU (12), were restreaked on fluoro-anthranilate agar to confirm resistance. Growth inhibition experiments in broth revealed that all three mutants exhibited high-level resistance to fluoro-anthranilate (Table 1). As expected, targeted sequencing of fluoro-anthranilate-resistant M. bovis BCG revealed missense mutations in the N-terminal region of TrpE (Table 1). One strain (BCG_FA^R^_M1) harbored a mutation in the Trp allosteric binding motif _53_LLESX_10_ S_67_ (Fig. 5A), where Ser67 was replaced by Ala, likely abrogating Trp binding (14, 19). The second fluoro-anthranilate-resistant M. bovis BCG mutant (BCG_FA^R^_M2) harbored a Val130Phe mutation (Fig. 5A). Targeted sequencing of the fluoro-anthranilate-resistant *M. tuberculosis* strain (MTB_FA^R^_M1) (Table 1) revealed a His170Asn missense mutation in the _170_ HHEGT_174_ motif, which interacts with the allosteric binding motifs of both TrpE subunits as well as with its counterpart of the partner subunit (15) (Fig. 5A). In the crystal structure of homodimeric TrpE, His170 is located next to the allosteric X_10_ loop of the _53_LLESX_10_S_67_ Trp binding motif of its own subunit, where it interacts via hydrogen bonding with Arg65 located in the X_10_ loop (AENGRSWSR) (Fig. 5A) and via stacking with its symmetry mate, His170′, located in the _170_ HHEGT_174_′ motif of the partner monomer (15) (Fig. 5B). Thus, His170 mutations are likely to affect Trp binding to both TrpE partner subunits.

To confirm that the fluoro-anthranilate resistance mutations in the allosteric Trp pocket of TrpE indeed prevent Trp binding, we generated the respective recombinant TrpE proteins TrpE^S67A^ and TrpE^H170N^ (see Fig. S2 and S3 in the supplemental material) harboring the Ser67Ala substitution found in BCG_FA^R^_M1 and the His170Asn mutation present in strain MTB_FA^R^_M1, and carried out enzyme inhibition experiments as described previously (15) (see Fig. S4 in the supplemental material). As expected, the activity of recombinant wild-type TrpE was inhibited by the physiological inhibitor Trp, with a 50% inhibitory concentration (IC_50_) of 1.6 µM, consistent with previous reports (12, 15). This was also true for F-Trp, the transformation product of fluoro-anthranilate, which showed an IC_50_ of 4.1 µM. In contrast, the IC_50_ values for Trp and F-Trp against the recombinant TrpE harboring Ser67Ala or His170Asn mutations increased by more than 100-fold (Fig. S4). These results show that fluoro-anthranilate-associated resistance mutations in the allosteric pocket of TrpE indeed abrogate Trp (and F-Trp) binding and are consistent with data generated for the fluoro-anthranilate-resistant Phe67Ile mutation in the Trp binding pocket of TrpE reported by Zhang and colleagues (12).

10.1128/mBio.02781-18.3 FIG S2 SDS-PAGE of purified recombinant TrpE proteins. His-tagged recombinant wild-type (WT) and Ser67Ala (S67A), and His170Asn (H170N) TrpE proteins were expressed in E. coli BL21 and purified. Samples were prepared by mixing with 4× NuPAGE lithium dodecyl sulfate (LDS) sample buffer (Novex) and 10 mM reducing agent dithiothreitol (DTT). Reduced samples were subjected to 4 to 12% NuPAGE Bis-Tris precast polyacrylamide gel (Life Technologies, catalog no. NP0301BOX) using 1× MES (morpholineethanesulfonic acid) buffer (Life Technologies, catalog no. NP0002) according to the manufacturer’s protocol and stained with Coomassie blue. One major band for each protein preparation migrating at the expected molecular weight (calculated molecular weight of 6× His-tagged TrpE = 56.6 kDa) was observed. The identity of the band was confirmed by in-gel digestion using an Applied Biosystems 4800 proteomics analyzer (Fig. S3). Download FIG S2, TIF file, 0.4 MB. Copyright © 2019 Negatu et al. 2019 Negatu et al. This content is distributed under the terms of the Creative Commons Attribution 4.0 International license.

10.1128/mBio.02781-18.4 FIG S3 Mascot search results of in-gel digestion. In-gel digestion was analyzed by MALDI-TOF MS. The highest score was observed for *M. tuberculosis* TrpE: scores higher than 81 are considered significant (48). Download FIG S3, TIF file, 0.2 MB. Copyright © 2019 Negatu et al. 2019 Negatu et al. This content is distributed under the terms of the Creative Commons Attribution 4.0 International license.

10.1128/mBio.02781-18.5 FIG S4 Enzymatic activity and Trp and F-Trp inhibition experiments of recombinant wild-type and S67A and H170N mutant TrpE proteins. (A) Enzymatic activity of recombinant TrpE proteins. (B, C, and D) Effect of Trp and F-Trp on the enzymatic activity of the recombinant wild-type (B) and S67A (C) and H170N mutant (D) TrpE proteins. A dose-dependent inhibition of wild-type TrpE was fitted to determine IC_50_ values of 1.6 ± 0.01 and 4.1 ± 0.01 μM for Trp and F-Trp, respectively. The mutant TrpE proteins were insensitive to Trp and F-Trp, with more than 100-fold resistance. Experiments were carried out three times independently in duplicate. Mean values and standard deviations are shown. Download FIG S4, TIF file, 0.1 MB. Copyright © 2019 Negatu et al. 2019 Negatu et al. This content is distributed under the terms of the Creative Commons Attribution 4.0 International license.

Next, we determined whether fluoro-anthranilate-resistant mutations conferred resistance to IPA as predicted. Table 1 shows that all three strains harboring fluoro-anthranilate-resistant TrpE mutations indeed showed high-level cross-resistance to IPA. Direct biochemical IPA inhibition experiments with recombinant proteins could not be carried out because of strong autofluorescence of IPA and the resulting assay interference (12) (see Fig. S5 in the supplemental material).

10.1128/mBio.02781-18.6 FIG S5 Dose response of IPA autofluorescence. RFU, relative fluorescence units. Download FIG S5, TIF file, 0.1 MB. Copyright © 2019 Negatu et al. 2019 Negatu et al. This content is distributed under the terms of the Creative Commons Attribution 4.0 International license.

### Expression of *trpE* alleles with altered TrpE binding site in the wild-type background confer IPA resistance.

**FIG 6:**
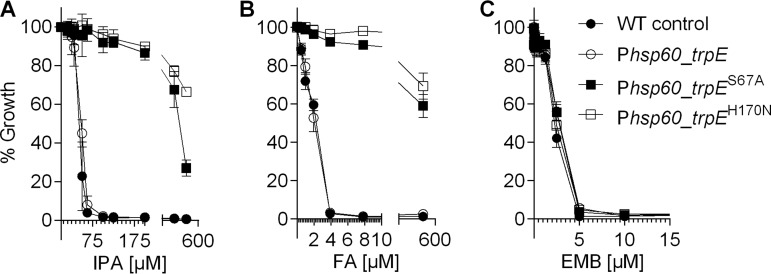
Effect of expression of *trpE* alleles Ser67Ala and His170Asn in the wild-type M. bovis BCG background on IPA’s growth-inhibitory activity. (A) Exponentially growing cultures of M. bovis BCG harboring plasmid pMV262, containing the coding sequence of TrpE^Ser67Ala^, TrpE^His170Asn^, and the TrpE wild type expressed constitutively from the plasmid’s *hsp60* expression signals, and “empty” pMV262 vector (“WT control”) were treated with increasing IPA concentrations, and the OD_600_ was measured after 7 days. (B) Experiment as in panel A, but using increasing concentrations of the positive control fluoro-anthranilate (FA) instead of IPA. (C) Experiment as in panel A but using the non-Trp synthesis-targeting negative-control drug ethambutol (EMB). Experiments were carried out three times independently in duplicate. Mean values and standard deviations are shown.

If the mutant *trpE* alleles Ser67Ala and His170Asn cause IPA resistance and do so by preventing allosteric inhibition of TrpE by IPA, these alleles should be dominant, i.e., they should confer IPA resistance when expressed in *trpE* wild-type background. To test this prediction, we isolated the *trpE* Ser67Ala and His170Asn alleles via PCR (see Table S1 in the supplemental material) and expressed these genes in M. bovis BCG wild type under the control of the p*hsp60*-based constitutive expression system supplied by the episomal plasmid pMV262 (26). We then determined the effect of *trpE* heterozygosity on the growth-inhibitory activity of IPA. Figure 6A shows that expression of both mutant *trpE* alleles caused IPA resistance. As expected, expression of the mutant *trpE* alleles also caused decreased susceptibility to the positive control, fluoro-anthranilate (Fig. 6B), but did not affect susceptibility of BCG to the negative control, ethambutol (Fig. 6C), suggesting that expression of mutant *trpE* alleles does not cause general drug resistance. Expression of the wild-type *trpE* allele did not affect susceptibility to IPA or fluoro-anthranilate as expected (13) (Fig. 6A and B). Together, these results show that TrpE amino acid substitutions Ser67Ala and His170Asn in M. bovis BCG and *M. tuberculosis* are responsible for the observed resistance to IPA and fluoro-anthranilate and confirm the expected dominant phenotype of these mutations.

10.1128/mBio.02781-18.8 TABLE S1 Primers used in the study. Download Table S1, PDF file, 0.1 MB. Copyright © 2019 Negatu et al. 2019 Negatu et al. This content is distributed under the terms of the Creative Commons Attribution 4.0 International license.

### Selection of spontaneous IPA-resistant mutants identifies two *trpE*-independent mechanisms of IPA resistance.

To determine whether allosteric inhibition of TrpE is the major and/or only mechanism of action of IPA, M. bovis BCG and *M. tuberculosis* cultures were plated on IPA agar, and resistant colonies were restreaked on IPA agar to confirm resistance. Interestingly, the spontaneous IPA resistance mutation frequency was about 5 × 10^−6^/CFU, more than 100-fold higher than the frequency of resistance observed for fluoro-anthranilate, suggesting that additional, non-*trpE*-related resistance mechanisms may exist.

Indeed, MIC determinations of six IPA-resistant M. bovis BCG strains (BCG_IPA^R^_M1 to -M6) showed low-level IPA resistance compared to the high-level resistance displayed by *trpE* mutants BCG_FA^R^_M1/M2 and MTB_FA^R^_M1 selected on fluoro-anthranilate (Table 1). Furthermore, these low-level IPA-resistant mutants were fluoro-anthranilate sensitive. Interestingly, one IPA-resistant strain (BCG_IPA^R^_M7) displayed a third drug susceptibility pattern: high-level IPA resistance (similar to BCG_FA^R^_M1, M2 and MTB_FA^R^_M1 selected on fluoro-anthranilate) combined with a low level of resistance to fluoro-anthranilate. As expected, targeted sequencing showed no polymorphisms in *trpE*.

MIC determination of six IPA-resistant *M. tuberculosis* strains (MTB_IPA^R^_M1 to -M6) showed a similar result. Four out of the six mutants (MTB_IPA^R^_M1 to -M4) showed low-level IPA resistance and were fluoro-anthranilate sensitive (Table 1). Two out of the six strains (MTB_IPA^R^-M5 and -M6) showed high-level IPA resistance and low-level resistance to fluoro-anthranilate, similar to BCG_IPA^R^_M7 (Table 1). Again, targeted sequencing showed no polymorphisms in *trpE*. These phenotypic characterization results suggested two additional, *trpE*-independent resistance mechanisms against IPA. Whereas *trpE*-dependent resistance mechanisms cause high-level IPA and fluoro-anthranilate resistance, the *trpE*-independent mechanisms confer either low-level IPA resistance with no fluoro-anthranilate resistance or high-level IPA resistance associated with low-level fluoro-anthranilate resistance.

To explore the genetic basis for the two *trpE*-independent resistance phenotypes, we subjected the six IPA-resistant *M. tuberculosis* strains (MTB_IPA^R^_M1 to -M6) to whole-genome sequencing. The four *M. tuberculosis* strains (MTB_IPA^R^_M1 to -M4) displaying low-level IPA and no fluoro-anthranilate resistance harbored various nonsense, deletion, and missense mutations in Rv0880 (Table 1). The Rv0880 gene encodes a nonessential transcription factor belonging to the family of multiple antibiotic resistance regulators (MarR) (27, 28), regulating expression of a 23-gene regulon (29) and influencing bedaquiline susceptibility (30). How Rv0880 mutations affect IPA susceptibility remains to be determined and is likely to involve an indirect mechanism (30).

The two *M. tuberculosis* strains MTB_IPA^R^-M5 and -M6 displaying high-level IPA and low-level fluoro-anthranilate resistance harbored missense mutations (Arg100Ser and Ser64Pro) in Rv0948c (Table 1). Rv0948c encodes a cytoplasmic chorismate mutase catalyzing the conversion of chorismate, the substrate of TrpE, to prephenate, the precursor for the biosynthesis of phenylalanine and tyrosine (31) (Fig. 2). Both missense mutations are located in the catalytic domain of the enzyme, and Arg100 was shown to be involved in its catalytic activity (32). Hence, it is conceivable that IPA-resistant mutations in Rv0948c reduce the catalytic activity of chorismate mutase, resulting in an increase in the concentration of the starter substrate of Trp biosynthesis, chorismate. This in turn may influence the vulnerability of the Trp pathway to inhibition of TrpE by IPA and fluoro-anthranilate (32, 33). Rather than being a direct target of IPA, Rv0948c appears to rewire the anabolism of aromatic amino acids, impacting vulnerability of the Trp biosynthetic pathway to inhibitors.

### Counterscreening of spontaneous IPA-resistant mutants for high-level fluoro-anthranilate resistance identifies *trpE* mutations.

The ∼100-fold difference in resistance frequency to IPA and fluoro-anthranilate (Table 1) led us to hypothesize that high-level fluoro-anthranilate cross-resistant mutants harboring lesions in *trpE* could be detected in a collection of spontaneous IPA-resistant mutants at a frequency of about 0.1 to 1%. To identify such high-level IPA–fluoro-anthranilate double-resistant mutants, we carried out a second IPA-resistant mutant selection experiment with *M. tuberculosis*, in which we isolated and confirmed 200 IPA-resistant colonies (Table 1). Then we determined cross-resistance to fluoro-anthranilate and identified five IPA–fluoro-anthranilate double-resistant colonies. Broth MIC determinations of these five strains revealed four high-level IPA–high-level fluoro-anthranilate-resistant strains (MTB_IPA^R^_M7 to -M10), suggesting that these strains may harbor *trpE* mutations. Targeted sequencing of these four high-level double-resistant strains showed indeed missense mutations at various locations in the N-terminal part of TrpE around the Trp allosteric binding motif (Fig. 5 and Table 1). These mutations included again a substitution at His170, this time with Arg (MTB-IPA^R^_M8 to -M10) (Fig. 5 and Table 1). These results confirmed that high-level IPA–fluoro-anthranilate resistance is associated with missense mutations in the allosteric Trp binding site of TrpE. These mutations can be obtained via selection on fluoro-anthranilate- or IPA-containing medium.

Whereas four out of the five isolated IPA–fluoro-anthranilate double-resistant strains showed high-level IPA and high-level fluoro-anthranilate resistance, one strain (MTB_IPA^R^_M11) showed high-level IPA and low-level fluoro-anthranilate resistance (Table 1), suggesting that this strain may harbor a mutation in the chorismate mutase gene, Rv0948c. Indeed, sequencing showed that this strain harbored a *trpE* wild-type gene and a missense mutation in the chorismate mutase gene (Table 1). This time the polymorphism was located at codon 33, resulting in a substitution of Arg by Leu. Arg33 of chorismate mutase is conserved across different species of bacteria and predicted to be involved in substrate binding (32). Thus, this mutation may affect activity of the enzyme.

Among the 200 resistant mutants isolated on IPA, 195 had shown no fluoro-anthranilate resistance. To characterize the resistance phenotype of these fluoro-anthranilate-sensitive strains, we randomly selected seven strains (MTB_IPA^R^_M12 to -M18) and determined their MICs. All seven strains showed low-level IPA resistance and no fluoro-anthranilate resistance in liquid medium, suggesting that their resistance is due to mutation in the transcription factor gene Rv0880. Sequencing indeed confirmed that all 7 strains harbored polymorphisms in Rv0880 (Table 1). We also subjected the M. bovis BCG mutants BCG_IPA^R^_M1 to -M7 (Table 1) to targeted sequencing of the Rv0948c and Rv0880 orthologues. As expected, the high-level IPA–low-level fluoro-anthranilate-resistant strain (BCG_IPA^R^_M7) harbored a mutation in the chorismate mutase gene (Rv0948c) (Table 1). Consistent with the corresponding *M. tuberculosis* observations, the remaining low-level IPA-resistant–fluoro-anthranilate-susceptible strains displayed polymorphism in the orthologue of the transcription factor Rv0880 (Table 1).

Taken together, these results show that IPA resistance can emerge via at least three different mechanisms (Table 2): (i) mutations in TrpE abrogating feedback inhibition, (ii) mutations likely to reduce chorismate mutase activity, thereby reducing vulnerability of the Trp pathway, and (iii) mutations in transcription factor Rv0880, which appears to be involved in general drug resistance.

**TABLE 2:** Summary of IPA–fluoro-anthranilate (FA) susceptibility patterns, their associated genotypes, and proposed mechanisms of resistance

IPA/FA susceptibility pattern	Gene harboring polymorphisms, gene product	Type of mutations and their location	Proposed mechanism of IPA/FA resistance
High IPA/FA resistance	*trpE*, anthranilate synthase	Missense mutations at allosteric Trp binding site	Loss of IPA/F-Trp^*a*^ binding to allosteric Trp binding site; loss of TrpE inhibition
High IPA/low FA resistance	Rv0948c, chorismate mutase	Missense mutations at active site	Increased chorismate concentration due to reduced chorismate mutase activity decreases vulnerability of Trp pathway to IPA and F-Trp inhibition
Low IPA resistance/ FA sensitive	Rv0880, multiple antibiotic resistance regulator family transcription factor	Deletions and nonsense and missense mutations throughout coding sequence	To be determined; indirect effect on IPA susceptibility via changes in expression of respective regulon members

a The Trp biosynthesis pathway coverts FA into F-Trp (20). For details, refer to Table 1.

### IPA is active against drug-resistant clinical *M. tuberculosis* isolates and nontuberculous mycobacteria.

The identification of the mechanism of action of IPA paves the way for a chemistry driven optimization program to generate more potent analogs. We showed that IPA is active against the drug-susceptible laboratory *M. tuberculosis* strain H37Rv. To ensure that anti-*M. tuberculosis* activity is not a laboratory strain artifact, we tested and confirmed activity of IPA against a collection of clinical *M. tuberculosis* isolates, including drug-resistant strains (Table S2).

10.1128/mBio.02781-18.9 TABLE S2 Activity of IPA against drug-resistant clinical *M. tuberculosis* isolates. Download Table S2, PDF file, 0.1 MB. Copyright © 2019 Negatu et al. 2019 Negatu et al. This content is distributed under the terms of the Creative Commons Attribution 4.0 International license.

In addition to *M. tuberculosis*, environmental mycobacteria (also called nontuberculous mycobacteria [NTM]) are increasingly causing difficult-to-cure lung disease (34). To determine whether IPA or its future analogs hold the potential for treating NTM infections in addition to tuberculosis, we determined whether IPA shows activity against these relatives of the tubercle bacillus. Growth inhibition experiments showed a spectrum of IPA susceptibilities in this group of mycobacteria, with Mycobacterium avium, Mycobacterium kansasii, and Mycobacterium chelonae showing susceptibility similar to *M. tuberculosis* and Mycobacterium fortuitum, Mycobacterium abscessus, and the mycobacterial model organism Mycobacterium smegmatis showing weaker inhibition by IPA (see Fig. S6 in the supplemental material). IPA showed no activity against selected Gram-positive and -negative bacteria (Fig. S6). Taken together, IPA appears to display broad-spectrum antimycobacterial-specific activity.

10.1128/mBio.02781-18.7 FIG S6 Effect of IPA on growth of mycobacteria and Gram-positive and Gram-negative bacteria and their Trp binding motifs. (A) Sequence alignment of Trp binding motifs of various bacterial species. Gray background shows different amino acids with reference to the *M. tuberculosis* Trp binding motif. (B and C) Susceptibility of exponentially grown *M. tuberculosis* and various nontuberculous mycobacteria (B) and representatives of Gram-positive and Gram-negative bacteria (C). Cultures were subjected to increasing concentrations of IPA, and the effect on growth was determined by OD_600_ measurements. The experiment was carried out three times independently. Means and standard deviations are shown. Download FIG S6, TIF file, 0.1 MB. Copyright © 2019 Negatu et al. 2019 Negatu et al. This content is distributed under the terms of the Creative Commons Attribution 4.0 International license.

## DISCUSSION

IPA is a host microbiota-derived metabolite that modulates mucosal immunity and exhibits neuroprotective effects (8, 35–40). IPA is produced from the aromatic amino acid tryptophan by several species residing in the gut, including Clostridium sporogenes (8). This low-molecular-weight metabolite can readily be detected in the human bloodstream and cerebrospinal fluid (7, 41). We recently discovered that IPA is active against *M. tuberculosis in vitro* and in a mouse model of TB infection (9). However, its bacterial target is unknown. Here, we have identified anthranilate synthase TrpE as the target of IPA in *M. tuberculosis*.

Since Trp is an allosteric inhibitor of mycobacterial TrpE required for Trp biosynthesis, we hypothesized that IPA, a structural analog of Trp, may exert its anti-*M. tuberculosis* activity by mimicking the Trp-mediated negative feedback on TrpE. Structural modeling and metabolic and chemical rescue, as well as genetic evidence, indicated that IPA indeed blocks Trp biosynthesis at the TrpE-catalyzed step and does so by binding to the allosteric pocket of Trp on TrpE. Resistance studies revealed three operating mechanisms. High-level resistance to IPA emerges by two distinct mechanisms in *M. tuberculosis*. Mutations in the allosteric binding site of Trp on TrpE eliminate not only negative-feedback control of Trp synthesis but also IPA inhibition of Trp synthesis. A second high-level IPA resistance mechanism involves metabolic rewiring. Missense mutations in the chorismate mutase, which utilizes the substrate of TrpE for the synthesis of other aromatic acids, appear to reduce the vulnerability of the Trp biosynthetic pathway to inhibition by small molecules. Finally, we uncovered a low-level IPA, likely an indirect resistance mechanism caused by mutations in the transcriptional regulator Rv0880 via an unknown mechanism.

We show that IPA is active not only against *M. tuberculosis*, including drug-resistant clinical isolates, but also against nontuberculous mycobacteria (NTM), such as Mycobacterium avium. Similar to multidrug-resistant tuberculosis, NTM lung disease is difficult to cure since first-line anti-TB drugs are largely inactive against common NTM species (34). Interestingly, IPA appears to display selective broad-spectrum antimycobacterial activity since it is inactive against major Gram-positive and Gram-negative pathogens, many of which harbor TrpE with a Trp allosteric binding site. The reasons behind this puzzling observation remain to be determined and could include drug uptake, efflux or metabolism (42), or differences in Trp binding motifs (Fig. S6). Although IPA did not show activity against representative nonmycobacterial microbes in our study, it is interesting to note that Mandelbaum-Shavit et al. reported antibacterial activity of the molecule against Legionella pneumophila causing pneumonia (Legionnaires’ disease) (43).

In conclusion, we find that IPA blocks tryptophan biosynthesis in *M. tuberculosis*. Intriguingly, IPA blocks this pathway by mimicking tryptophan, the physiological allosteric inhibitor of TrpE, the enzyme catalyzing the first committed step. Thus, IPA exploits a bacterial regulatory feedback mechanism to exert its antibacterial whole-cell activity. The present results, together with IPA’s proven *in vivo* activity, validate TrpE as a vulnerable target and reveal IPA as an attractive starting point for the discovery of a broad-spectrum antimycobacterial. The determination of TrpE as the target of IPA will enable the rational, target-based generation of a semisynthetic, next-generation IPA with improved potency.

## MATERIALS AND METHODS

### Docking studies.

The docking studies were performed using the Autodock 4.2.6 (AD4) and AutoDock Vina 1.1.2 (Vina) software (17, 18). The crystal structure of anthranilate synthase component I (TrpE) from *M. tuberculosis* was retrieved from the Protein Data Bank (PDB no. 5CWA) (15). The TrpE protein was prepared using the MOE2016.0802 (MOE) software (44). Issues with alternate or missing side chains, chain breaks, and terminal residues were corrected. Cocrystallized ions, water, and ligands were deleted. Hydrogens were added to the target structure using the Protonate3D module in MOE to predict the rotamers and protonation and tautomeric states of each residue at pH 7. The protein structure was saved in the PDB format. The 3D structures of IPA and l-Trp were retrieved from PubChem database in the SDF format. The structure of l-Trp was further optimized using the MMFF94x force field in MOE, and both structures were saved in the mol2 format. The protein, ligand, grid parameter, and docking parameter files were prepared using AutoDockTools 1.5.6. Kollman and Gasteiger charges were assigned to the protein and ligand residues, respectively. The grid box was set to cover most of the protein spaces, including the chorismate and Trp binding sites, with dimensions of 48.6 by 48.6 by 44.8 Å. For AD4, the docking was performed by setting the number of evaluations and number of Lamarckian genetic algorithm runs to 2,500,000 and 20, respectively. For Vina, the docking was performed by setting both the exhaustiveness and maximum number of binding modes to 20. The simulation was repeated 5 times for each ligand using AD4 or Vina. After the docking was completed, the output files were converted to SDF and TXT files and were analyzed using MOE.

### Bacterial strains, media, and cultivation.

M. tuberculosis H37Rv (ATCC 27294), M. bovis BCG Pasteur (ATCC 35734), M. smegmatis mc^2^155 (ATCC 700084), M. avium (ATCC 35717), M. abscessus (ATCC 19977), M. chelonae (ATCC 19539), M. fortuitum (ATCC 6841), Staphylococcus aureus (ATCC 12600), Escherichia coli (ATCC 25922), Pseudomonas aeruginosa (ATCC 27853), and Acinetobacter baumannii (ATCC BAA-2093) were obtained from the American Type Culture Collection. *Mycobacterium* species were grown in Middlebrook 7H9 (Becton Dickinson, Difco) complete broth medium supplemented with 0.05% Tween 80, 0.2% glycerol, and 10% albumin-dextrose-catalase (ADC) at 37°C. All other bacteria were grown in LB broth at 37^°^C with 200-rpm shaking. Clinical *M. tuberculosis* isolates (cMtb_1, cMtb_2, cMtb_3, and cMtb_4) were provided by the strain collection of the BSL3 core facility of the National University of Singapore. All experiments involving *M. tuberculosis* were performed in the BSL3 core facility of the National University of Singapore.

### Multiple-sequence alignment.

The amino acid sequence of TrpE for bacterial species were downloaded from UniProt. The sequence alignments were performed using the Clustal Omega sequence alignment tool of EMBL-EBI (https://www.ebi.ac.uk/Tools/msa/clustalo/).

### Chemicals and drugs.

All chemicals and drugs were purchased from Sigma-Aldrich, USA, unless indicated otherwise. 3-Indole propionic acid (catalog no. 220027), 5-fluoro-anthranilic acid (catalog no. 367982), and anthranilic acid (catalog no. A89855) were dissolved in 100% dimethyl sulfoxide (DMSO) at 400 mM. l-Tryptophan (catalog no. T0254), chorismic acid (catalog no. C1761), ethambutol (catalog no. E4630), and 5-fluoro-l-tryptophan (catalog no. AK-60150; Ark Pharm, Inc.) were dissolved in distilled water at 10 mM. All drugs were stored in aliquots at −20°C until use.

### Quantification of Trp metabolite.

To extract the intrabacterial Trp, we followed a previously published protocol (45). Briefly, exponentially grown M. bovis BCG cultures were adjusted to a final optical density at 600 nm (OD_600_) of 0.4. The cultures were treated with IPA, fluoro-anthranilate, or ethambutol with 0×, 0.5×, 1×, and 2× MICs and incubated at 37^°^C with 80-rpm shaking for 24 h. The following MICs were used under these culture conditions: IPA, 140 μM; fluoro-anthranilate, 6 µM; EMB, 3 µM. To measure growth of samples after 24 h of treatment, we transferred 100 µl of aliquots into a clear-bottom 96-well plate and measured the OD using a Tecan Infinite 200 Pro microplate reader. OD_600_ measurements after 24 h showed no significant differences. We collected pellets (∼10^9^ CFU/ml) of cultures by centrifugation at 3,200 rpm for 10 min and washing with 1× phosphate-buffered saline (PBS). The pellet was resuspended with 500 μl of 1× PBS and transferred into lysing matrix B tubes (MP Biomedicals). The samples were subjected to bead beating (Precellys 24 homogenizer) at 6,500 rpm three times for 30 s each as described earlier (45). The lysate was pelleted by centrifugation at 13,000 rpm for 10 min, and the supernatant was carefully transferred to new tubes for Trp quantification. Trp quantification was performed using liquid chromatography coupled to tandem mass spectrometry (LC-MS/MS [details are provided in Text S1 in the supplemental material]).

10.1128/mBio.02781-18.1 TEXT S1 Supplemental methods. Download Text S1, DOCX file, 0.1 MB. Copyright © 2019 Negatu et al. 2019 Negatu et al. This content is distributed under the terms of the Creative Commons Attribution 4.0 International license.

### MIC determination.

MIC determinations were performed in 96-well plates (Corning catalog no. 3527) as described previously (9). Briefly, an inoculum of exponentially growing *M. tuberculosis* was adjusted to a final density of ∼10^6^ CFU/ml in Middlebrook 7H9 (Becton Dickinson) complete broth medium. Serially diluted drug concentrations were mixed with the inoculum in a 96-well plate and incubated at 37^°^C with orbital shaking at 80 rpm for 7 days. Turbidity at 600 nm was measured using a Tecan Infinite 200 Pro microplate reader. Percentage of inhibition was calculated relative to the drug-free samples. Regarding non-*M. tuberculosis* mycobacterial species, exponentially growing M. smegmatis, M. avium, M. abscessus, M. chelonae, and M. fortuitum cells were diluted in Middlebrook 7H9 complete medium to a final inoculum density of ∼10^6^ CFU/ml and incubated with IPA in 96-well flat-bottom microtiter plates for 2, 5, 3, 4, and 4 days, respectively. Exponentially growing S. aureus, E. coli, Pseudomonas aeruginosa, and Acinetobacter baumannii cells were diluted in LB medium and incubated with IPA overnight. Ten-point 2-fold serial dilutions of IPA starting from a concentration of 1 mM were used as described previously (9).

### Chemical supplementation studies.

To evaluate the effect of anthranilate and Trp supplementation on the activity of IPA, we tested IPA against *M. tuberculosis* in 7H9 complete medium with or without supplementation with 1 mM Trp or 0.2 mM anthranilate. The assay was performed in 96-well plates as described for MIC determination. The OD measurements were complemented by an agar growth assay. *M. tuberculosis* culture samples were transferred from 96-well plates onto single-well 7H11 complete agar microplate (Stem Corporation, Japan) using a 96-well replicator (Stem Corporation, Japan). The plates were incubated for 10 days at 37°C prior to visual examination and photo taking.

### Spontaneous resistant mutant selection.

Mutant generation was performed as described previously (46). Briefly, 10^6^ to 10^9^ CFU from exponentially growing *M. tuberculosis* or M. bovis BCG cultures were plated on complete 7H11 agar containing 500 or 750 µM IPA or 150 µM fluoro-anthranilate. The plates were incubated for 4 weeks at 37°C. Mutant colonies were colony purified and confirmed for resistance by restreaking on agar containing the same concentration of drug used for selection. MIC determinations were performed to determine the level of resistance. To select fluoro-anthranilate cross-resistant IPA mutants, we inoculated 200 IPA-resistant colonies in 96-well plates containing 100 µl of 7H9 broth medium with 150 µM fluoro-anthranilate. Bacteria in wells showing growth were restreaked on 7H11 agar medium containing 150 µM fluoro-anthranilate. The plates were incubated for 4 weeks at 37°C. Isolated single colonies were inoculated into 10 ml of 7H9 complete broth medium, and cross-resistance was confirmed by performing MIC determinations with the wild-type strain as the control.

### Targeted and whole-genome sequencing.

Extraction of genomic DNA was performed as described previously using the phenol-chloroform method (45, 47). To perform targeted sequencing, PCR amplification was performed using custom-designed primers (Table S1) following instructions provided by a high-fidelity PCR kit (Toyobo no. F1066K). Sanger sequencing was performed for two independent PCR products by Bio Basic Asia Pacific, Singapore. For whole-genome sequencing (WGS), library construction, whole-genome sequencing, and bioinformatics analyses were performed by NovogeneAIT Genomics, Singapore (Details of WGS and bioinformatics analysis are described in Text S1.) Following whole-genome sequencing, Sanger sequencing was performed to confirm mutations in *trpE*, Rv0880, and Rv0948c amplified by custom-designed primers (Table S1).

### TrpE expression studies.

To overexpress *trpE* (Rv1609), we cloned the gene into the pMV262 episomal plasmid under the control of the *hsp60* constitutive promoter (26). First, the gene was amplified from the wild-type or mutant genomic DNA of *M. tuberculosis*/M. bovis BCG using custom primers (Table S1). The amplification was performed according to the KOD-plus-neo high-fidelity PCR kit protocol (Toyobo no. F1066K). The PCR products were purified by the QIAquick PCR purification kit (Qiagen, Hilden, Germany) and digested along with pMV262 vector using 3 μl of HindIII-HF (New England Biolabs no. R3104S) for 3 h at 37°C. The digested products were purified by agarose gel electrophoresis. Prior to ligation, the vector was treated with 0.5 μl of alkaline phosphatase (CLP; New England Biolabs no. M0690S) for 30 min to prevent religation. Following overnight ligation at 16^0^C, the ligation products were transformed into competent E. coli DH5α cells. Transformants were confirmed by colony PCR, restriction digestion, and Sanger sequencing (Bio Basic Asia Pacific, Singapore). These constructs were electroporated into M. bovis BCG strains, which were plated on kanamycin (25 μg/ml)-containing 7H11 agar plates and incubated at 37°C for 3 weeks. Positive colonies were confirmed by restreaking on kanamycin-containing agar plates. Verified single colonies were inoculated into 7H9 complete broth medium, and stocks were made in 10% glycerol and stored at −80^°^C until use. Transformation of mutant constructs pMV262-*phsp60*-*trpE* (Ser67Ala or His170Asn) in the E. coli DH5α strain consistently failed. Thus, we directly electroporated the ligation products into M. bovis BCG, where transformants could be obtained. Positive clones were verified by colony PCR, restriction digestion, and Sanger sequencing (Bio Basic Asia Pacific, Singapore).

### Recombinant His tag TrpE generation.

Cloning, expression, and purification of TrpE were performed as described previously (16). Briefly, the *trpE* gene (Rv1609) was amplified from the genomic DNA of wild-type or mutant *M. tuberculosis*/M. bovis BCG using custom primers (Table S1). The amplification was performed according to the KOD-plus-neo high-fidelity protocol (Toyobo no. F1066K). The PCR products were purified by QIAquick PCR purification kit (Qiagen, Hilden, Germany). The purified PCR products and pET30a+ vector were digested using 2 µl of NcoI-HF (20,000 U/ml) and HindIII-HF (20,000 U/ml) in 1× CutSmart buffer (New England BioLabs no. B7204S) for 3 h. Following overnight ligation at 16^°^C using T4 DNA ligase, the products were transformed into the competent E. coli BL21(DE3) strain and plated on kanamycin (25 µg/ml)-containing LB agar overnight at 37°C. Positive colonies were confirmed by colony PCR, restriction digestion, and Sanger sequencing (Bio Basic Asia Pacific, Singapore).

To express the recombinant protein, a single colony was inoculated in 10 ml of LB medium containing 25 μg/ml kanamycin overnight at 37°C. The following day, 5 ml of overnight culture was inoculated into 1 liter LB medium containing 25 μg/ml kanamycin and incubated until the OD_600_ reached ∼0.5 at 37°C. TrpE expression was induced by the addition of 0.02 mM isopropyl-β-d-thiogalactopyranoside (IPTG) as described previously (16). The cultures were incubated for an additional 16 h at 20°C. The protein purification was performed according to the Qiagen Ni-nitrilotriacetic acid (NTA) Superflow column protocol (Qiagen, Hilden, Germany). The purified proteins were subjected to buffer exchange with 5 mM Tris-HCl (2.5% glycerol) and concentrated using an Amicon Ultra-15 filter device by centrifugation at 4,000 × *g* for 20 min at 4^°^C (Millipore Corporation, Bedford, MA). The proteins were stored at −80^°^C until use. The molecular weight of His-TrpE was estimated by Coomassie brilliant blue R250-stained sodium dodecyl sulfate-polyacrylamide gel electrophoresis (SDS-PAGE) with molecular weight standards (calculated mass of TrpE including the 6× His tag, 56.6 kDa). In-gel digestion was performed to confirm the protein using an Applied Biosystems 4800 proteomics analyzer for matrix-assisted laser desorption ionization–time of flight tandem mass spectrometry (MALDI-TOF/TOF) (Applied Biosystems, Framingham, MA), which provides the highest score, 1,310, confirming the protein is indeed TrpE (48, 49) (Fig. S3 and Text S1). This analysis was performed by the Protein and Proteomics Center, Department of Biological Sciences, National University of Singapore. The concentration of the proteins was measured following a colorimetric detection method using the bicinchoninic acid (BCA) kit (Thermo Scientific no. 23225).

### Biochemical TrpE studies.

To perform enzymatic assays, we used wild-type and Ser67Ala and His170Asn mutant versions of recombinant TrpE. To measure the activity of the enzymes, the rate of the anthranilate formation was monitored fluorometrically with λ_excitation_ of 320 nm and λ_emission_ of 460 nm using a coupled enzymatic assay. The concentration of anthranilate formed was determined from a standard curve established by the concentration of anthranilic acid versus its fluorescence intensity measured by a Tecan Infinite 200 Pro microplate reader. To determine the *K_m_* and *V*_max_ of the enzymes, various concentrations of chorismate were added to the reaction mixture (20 mM Tris [pH 9], 100 mM NH_4_Cl, 10 mM MgCl_2_, 0.1 mM EDTA, 0.075 mg/ml wild-type or mutant enzymes) in 96-well plates as described previously (15, 16). The *K_m_* and *V*_max_ for chorsimate were calculated by nonlinear curve fitting to the Michaelis-Menten equation using GraphPad Prism 6 as described previously (15). The *in vitro* enzymatic inhibition assay was performed in a final volume of 200 µl containing 100 mM NH_4_Cl, 10 mM MgCl_2_, 0.1 mM EDTA, 20 mM Tris (pH 9), 0.075 mg/ml wild-type and mutant TrpE proteins, and 50 µM chorismate in black 96-well plates as described previously (15). Trp and F-Trp were dissolved in water at a concentration of 10 mM and tested in the assay with concentrations ranging from 0.1 to 100 µM. Prior to the addition of the substrate chorismate, the mixture was incubated for 10 min at 25°C. The IC_50_ values were calculated as described previously (50). All assays were performed in triplicate.

### Drug susceptibility testing using Bactec MGIT 960.

To determine the activity of IPA against multidrug-resistant *M. tuberculosis* strains, the Bactec MGIT 960 system (Becton Dickinson, Sparks, MD) was used according to the manufacturer’s instructions and the MGIT manual by FIND (Geneva, Switzerland). We used four clinical *M. tuberculosis* isolates from the collection of the National University of Singapore BSL3 core facility and the laboratory strain H37Rv. Susceptibility testing for streptomycin (SM [1.0 μg/ml]), isoniazid (INH [0.1 μg/ml]), rifampin (RIF [1.0 μg/ml]), ethambutol (EMB [5.0 μg/ml]), and pyrazinamide (PZA [100 μg/ml]) was done with the MGIT AST SIRE and PZA kits. For IPA test tubes, 100 μl of IPA (final concentration of 28 μg/ml) was aseptically added into the MGIT tubes supplemented with 0.8 ml of Bactec 960 SIRE supplement.
