# Supplementary figures and images for: Gut Microbiota Metabolite Indole Propionic Acid Targets Tryptophan Biosynthesis in Mycobacterium tuberculosis
Source: mBio. 2019 Mar 26;10(2):e02781-18. doi: 10.1128/mBio.02781-18 (PMC6437058; doi:10.1128/mBio.02781-18)

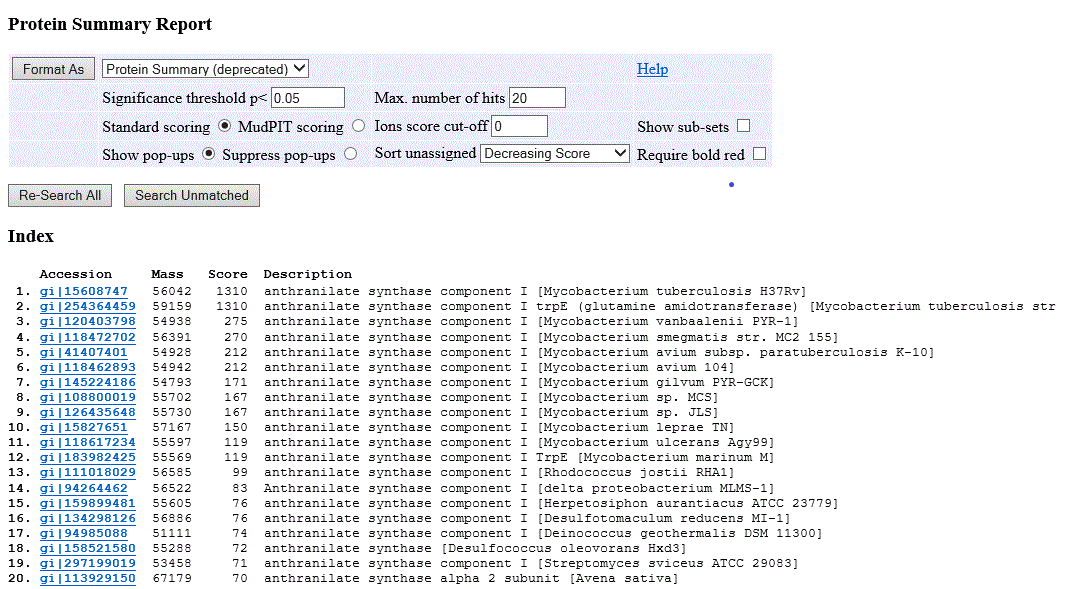

Supplement: FIG S3 [file mBio.02781-18-sf003.tif]
