# Supplementary material for: Gut Microbiota Metabolite Indole Propionic Acid Targets Tryptophan Biosynthesis in Mycobacterium tuberculosis
Source: mBio. 2019 Mar 26;10(2):e02781-18. doi: 10.1128/mBio.02781-18 (PMC6437058; doi:10.1128/mBio.02781-18)
Supplement: TABLE S1 [file mBio.02781-18-st001.pdf]

TABLE S1. Primers used in the study

Purpose	Primer name	Gene	Sequence (5' – 3')
Expression of *trpE* alleles in Mtb	TrpE-HindIII_F	*trpE*	CCG AAG CTT GTG CAC GCC GAC CTC GCA G
	TrpE-HindIII_R		CCG AAG CTT TTA GCA GCC ACT GCG GTT CGC G
Recombinant TrpE production	TrpE-NcoI_F	*trpE*	GGC CAT GGT GCA CGC CGA CCT CGC AG-3'
	TrpE-HindIII-R		CCG AAG CTT TTA TTA GCA GCC ACT GCG GTT CG
Sanger sequencing	TrpE_Mut_F	*trpE*	GCA GCG TCG CTA ACG CTT TGT CG
	TrpE_Mut-R		CGT GCG GCC ATC CAC AGC
	Rv0948c_Mut_F	Rv0948c	CGC AAT TGT GAA ACT CCA G
	Rv0948c_Mut_R		GCT CGT CTC TTG TGT GAG
	Rv0880_Mut-F	Rv0880	GAT GCC CTA GTC TGA ACT TCC G
	Rv0880_Mut-R		GGA TCG CAG ACG TCC TGA AC
