# Supplementary material for: Gut Microbiota Metabolite Indole Propionic Acid Targets Tryptophan Biosynthesis in Mycobacterium tuberculosis
Source: mBio. 2019 Mar 26;10(2):e02781-18. doi: 10.1128/mBio.02781-18 (PMC6437058; doi:10.1128/mBio.02781-18)
Supplement: TABLE S2 [file mBio.02781-18-st002.pdf]

TABLE S2. Activity of IPA against drug resistant clinical Mtb isolates

Clinical strains	Antimycobacterial drugs					
	INH (0.1 µg/ml)	RIF (1 µg/ml)	PZA (100 µg/ml)	EMB (5 µg/ml)	STR (1 µg/ml)	IPA (28 µg/ml)
H37Rv	S	S	S	S	S	S
cMtb_1	R	R	S	S	S	S
cMtb_2	R	R	R	S	S	S
cMtb_3	S	R	S	S	R	S
cMtb_4	R	S	R	R	S	S
