# Supplementary material for: Gut Microbiota Metabolite Indole Propionic Acid Targets Tryptophan Biosynthesis in Mycobacterium tuberculosis
Source: mBio. 2019 Mar 26;10(2):e02781-18. doi: 10.1128/mBio.02781-18 (PMC6437058; doi:10.1128/mBio.02781-18)
Supplement: TEXT S1 [file mBio.02781-18-s0001.docx]

**SUPPLEMENTAL METHODS**

**Whole genome sequencing and bioinformatics analysis**

The quality of the genomic DNA checked by a Nanodrop (Thermo Fisher Scientific) and quantified by Qubit (Thermo Fisher Scientific) prior to library construction. To prepare the library, a total amount of 1.0 μg DNA per sample was used as input material for the DNA sample preparations. Sequencing libraries were generated using Truseq Nano DNA HT Sample preparation Kit (Illumina USA) following manufacturer's recommendations and index codes were added to attribute sequences to each sample. The genomic DNA is randomly fragmented to a size of 350bp by Covaris cracker, then DNA fragments were end polished, A-tailed, and ligated with the full-length adapter for Illumina sequencing with further PCR amplification. At last, PCR products were purified (AMPure XP system) and libraries were analysed for size distribution by Agilent2100 Bioanalyzer and quantified using real-time PCR. The qualified libraries are fed into HiSeq/MiSeq sequencers after pooling according to its effective concentration and expected data volume. The sequenced reads were filtered by removing adaptors, reads containing N > 10% (N represents the base cannot be determined), and reads containing low quality (Qscore<= 5) base.

The fasta, genbank and genome annotation files for the reference strains, *M. bovis* BCG Pasteur 1173P2 (NC_008769.1) and *M. tuberculosis* H37Rv (NC_000962.3), were obtained from NCBI. Analysis was performed using two pipelines: In one pipeline, paired reads were mapped to the reference using bowtie2. The resulting SAM file from the mapping was converted to a BAM file using sam tools and the file was sorted and indexed. Mpileup was used to generate a BCF file which was converted to a variant call file (VCF) using bcf tools. In the second pipeline, paired end reads were mapped to the reference using bwa (version 0.7.10) and the resulting SAM file from the mapping was converted to a BAM file using Sam tools and the file was sorted and indexed. Mpileup was used to create a pileup file and variants (SNP and INDELs) were called using VarScan (version 2.3.6) with mileup2snp and mpileup2indel, respectively, and p-value threshold of 0.05. In both pipelines, the vcf file was converted to an annovar file using the convert2annovar perl script. Finally, the annovar file was compared to the annotation file from the reference genome to determine location and relevance of the variants using the annotate variation perl script. Annovar variation results file generated in each pipeline were combined using an in-house perl script and imported into Excel for analysis. To determine mutations involved in IPA resistance, only nonsynonymous mutations, insertions and deletions, satisfying the threshold criteria (Quality>30, Frequency>75%, Coverage >20), and not found in our wild type strain were included for the final analysis.

**MALDI-TOF/TOF In-gel protein identification methods**

In-gel protein identification was performed to confirm TrpE. The protein band (~ 2 mm) from the SDS-PAGE was excised and placed into the wells of SepPak tC18 u Elution Plate (Cat#186002318; Waters Pacific Pte Ltd). 100 µL of 50 mM ABC / 50% EtOH was added into each well and incubated for 20 min at room temperature. The gel was dehydrated by adding 100 µL of 100% EtOH to each well and incubated for 15 min at room temperature. To reduce the protein, 200 µL of 10 mM DTT/50 mM ABC was added to the wells and incubated for 1 hour at 56^0^C. 200 µL of 55 mM iodoacetamide/50 mM ABC was added into each well and incubated for 30 min at room temperature. 200 µl of 50 mM ABC was added into each well and incubated for an additional 15 min at room temperature. To wash and dehydrate the protein, 100 µl of 25 mM ammonium bicarbonate (ABC) /5% acetonitrile was added into each well and incubated for 30 min. Plate cover was removed and full vacuum (15–20" Hg) were applied. The vacuum was released once all the solution has been emptied from the wells. Then, 100 µl of 25mM ABC / 50% acetonitrile was added into each well and incubated for 30 min. After the buffer was removed, 200 µl of 100% acetonitrile was added into each well containing gel pieces and incubated for 10 min. Full vacuum was applied for 2 min to completely remove acetonitrile from the wells. To digest the protein, 15 µl of the prepared Trypsin (Sequencing Grade, Promega) Solution (10 ng/ul in 25 mM ABC buffer) was added into each well and incubated for 3 h at 37^0^C. To extract and wash the digested product, 8 µl of 100% acetonitrile were directly added onto the resin and incubated for 15 min at 37^0^C. Then, 130 µl of extraction/wash solution (1.0% formic acid for ESI MS) were added into each well and incubate at room temperature for 30 min. 100 µl of the extraction/wash Solution were added. To elute, 20 µl of elution solution were added in the center of the wells containing gel pieces. Vacuum applied to elute the peptides into a microtiter receiver plate. Samples spots were analyzed on the Applied Biosystems 4800 Proteomics Analyzer MALDI-TOF/TOF (Applied Biosystems, Framingham, MA, USA). 0.5 μl of the sample were spotted on a 384 well target plate and crystallized with 0.5 μl of CHCA (a-cyano-4-hydroxycinnamic acid) matrix solution (0.1% TFA, 50% ACN in MilliQ water, 5mg/ml). The instrument was calibrated with the premixed calibration peptide mixture and sample requisition process was done in following instrument setting (Operation Mode: Reflector; Delayed Extraction time: 25-1000 ns; Instrument mode: Positive Ion mode; Accelerating Voltage: 20000V; Mass Range: 450 to 1200 M/z; Laser power: 2900 for MS; 3200 for MS/MS; Laser shot frequency: 200 Hz; For MS mode: 1000 laser shots = 1 MS spectrum (50 shots /sub-spectrum); For MS/MS mode: 7500 laser shots = 1 MS/MS spectrum (50 shots /sub-spectrum); Collision Gas for CID: Nitrogen; Collision Energy: 1KV).

**LC/MS-MS analytical methods**

To create standard curves and quality control (QC) spiking solutions, neat 1 mg/ml stocks of tryptophan, solubilized in water, was serially diluted in 1x PBS pH 7.4 with 0.1% Tween 80. 20 µl of standards, QCs, blanks, and cell lysates were extracted by adding 200 µl of Acetonitrile/Methanol 50/50 protein precipitation solvent containing the internal standard (10 ng/ml Verapamil). Extracts were vortexed for 5 min and centrifuged at 4000 RPM for 5 min. 100 µl or supernatant was transferred for HPLC-MS/MS analysis and diluted with 100 µl of Milli-Q deionized water. LC/MS-MS quantitative analysis for tryptophan was performed on a Sciex Applied Biosystems Qtrap 6500+ triple-quadrupole mass spectrometer coupled to a Shimadzu 30ACMP HPLC system, and chromatography was performed on an Agilent Zorbax SB-C8 column (2.1x30 mm; particle size, 3.5 µm) using a reverse phase gradient elution. Milli-Q deionized water with 0.1% formic acid was used for the aqueous mobile phase and 0.1% formic acid in acetonitrile for the organic mobile phase. Multiple-reaction monitoring of parent/fragment transitions in electrospray positive-ionization mode was used to quantify all molecules. MRM transitions of 205.10/146.00 and 455.40/165.20 were used for tryptophan and Verapamil respectively. Sample analysis was accepted if the concentrations of the quality control samples and standards were within 20% of the nominal concentration. The level of quantitation for tryptophan was 1 ng/ml in lysate. Data processing was performed using Analyst software (version 1.6.2; Applied Biosystems Sciex).
